# Chemometric Classification and Bioactivity Correlation of Black Instant Coffee and Coffee Bean Extract by Chlorogenic Acid Profiling

**DOI:** 10.3390/foods13244016

**Published:** 2024-12-12

**Authors:** Yumei Chen, Wei Yu, Yuge Niu, Wenchen Li, Weiying Lu, Liangli (Lucy) Yu

**Affiliations:** 1 Institute of Food and Nutraceutical Science, Department of Food Science and Technology, School of Agriculture and Biology, Shanghai Jiao Tong University, Agriculture and Biology Building, 800 Dongchuan Road, Shanghai 200240, China; c18324939875@163.com (Y.C.); yu-wei@sjtu.edu.cn (W.Y.); yugeniu@sjtu.edu.cn (Y.N.); wensjtu@sjtu.edu.cn (W.L.); 2 Department of Nutrition and Food Science, University of Maryland, College Park, MD 20742, USA; lyu5@umd.edu

**Keywords:** phenolics, black instant coffee, ultra-performance liquid chromatography–mass spectrometry, chemometrics, cellular antioxidant activity

## Abstract

Chlorogenic acids (CGAs) play a key role in defining the quality and functionality of coffee products. CGA fingerprints of black instant coffee (BIC) and coffee bean extract (CBE) were profiled using ultra-performance liquid chromatography–mass spectrometry and analyzed by chemometrics. A total of 25 CGAs were identified. The BICs yielded higher levels of major CGAs than the CBEs. Furthermore, chemometrics methods, including principal component analysis (PCA) and partial least squares–discriminant analysis (PLS–DA), successfully classified the CBEs and the BICs. In vitro cellular antioxidant activity and viability assays between coffee products further confirmed the relationship between phenolic compounds and bioactivities. Compared to the CBEs, the BICs provided higher cellular toxicity and oxidant activity for hepatocellular carcinoma G2 (HepG2) cells. These results demonstrated that CGAs and their derivatives could be markers for studying coffee-related products. This study revealed the unique phenolic profiles of various coffee products, highlighting the differences between whole beans and soluble coffee.

## 1. Introduction

Coffee stands as a prominent and widely favored beverage, with global consumption rates being second only to water [1]. Consequently, coffee-related industries provide employment to approximately 100 million people worldwide [2]. The global popularity of coffee has increased significantly in recent years with the rise in globalization as well. China, for example, despite not being a traditional coffee-consuming country, has witnessed a surge in its coffee markets due to rapid economic growth and cultural shifts [3].

Diversified coffee products with different processing techniques exist nowadays due to consumer preferences. Soluble coffee products, including instant coffee, freeze-dried coffee, coffee concentrates, etc., are designed to quickly dissolve in hot water, providing a convenient experience. Although regular whole bean coffee may have a better taste, aroma, and texture, black instant coffee is more popular than whole bean coffee for its convenience [4]. Overall, statistics indicate that more exports of soluble coffee were achieved than whole bean coffee worldwide [5]. It is, therefore, important to evaluate the functional differences between whole bean coffee and soluble coffee.

Phenolic compounds are essential bioactive substances in coffee that have raised research interests. In particular, chlorogenic acids (CGAs) are a group of phenolic compounds that are widely distributed in plants, which play a significant role in determining the flavor, aroma, health functionalities such as antioxidant properties, and other quality attributes of coffee. For instance, the presence and abundance of CGAs influence the quality of a coffee brew, contributing to changes in its color, flavor, and aroma during the roasting process [6]. It is imperative to investigate the relationships between coffee quality and phenolic functionality, as well as their cellular antioxidant activity. On the other hand, different brewing and roasting processes induce physical and chemical changes in coffee beans [7]. Furthermore, phenolic acids, along with carbohydrates, tocopherols, homostachydrine, etc., can be used as specific chromatographic markers to distinguish the degrees of roasting and geographical origin or to detect potential adulteration [8]. Therefore, a comparative analysis of the phenolic profiles can provide scientific evidence of the functional properties of different coffee products.

Chemometrics is the application of mathematical and statistical methods to analyze chemical data, and it plays a crucial role in the analysis of complex datasets obtained from analytical chemistry techniques. In this study, we employed two chemometric techniques: principal component analysis (PCA) and partial least squares-discriminant analysis (PLS-DA). PCA transforms original variables into a new set of variables, called principal components, which are orthogonal and ordered such that the first few retain most of the variation present in all the original variables. PCA is used to visualize data in a lower-dimensional space, which helps in identifying patterns and relationships among samples. PLS-DA is a supervised technique that combines the predictive power of partial least-squares regression with the classification ability of discriminant analysis. PLS-DA finds linear combinations of the predictors that best separate the different classes of response variables.

Previous studies have focused on individual aspects of coffee chemistry, but a comprehensive comparison using analytical techniques coupled with chemometrics is lacking. This study aimed to profile the phenolic compositions of whole bean coffee and instant coffee products. Specifically, coffee bean extracts (CBEs) and black instant coffees (BICs) were processed and analyzed by ultra-performance liquid chromatography–mass spectrometry (UPLC-MS). The acquired fingerprints were further analyzed using chemometrics, including PCA, PLS-DA, and orthogonal PLS-DA (OPLS-DA). This allowed us to identify potential markers that could distinguish between different coffee products and allowed us to understand their functional properties. The different roasting degrees of whole bean coffee, as well as the compositional influence of cell toxicity and antioxidant activity on hepatocellular carcinoma G2 (HepG2) cells, were also investigated. The current study addressed a significant gap regarding the comparative analysis of phenolic profiles in whole bean coffee and instant coffee products. This work provided evidence as to whether phenols can serve as potential markers for whole bean coffee and black instant coffee. The chemometrics models also helped to identify candidate markers that may serve as a useful reference to evaluate the physiological functions of different forms of coffee products.

## 2. Materials and Methods

### 2.1. Materials and Chemical Reagents

Nine types of commercial coffee beans roasted to different degrees and nine types of instant coffee were purchased from local Chinese coffee distributors. Table S1 in the Supplementary Materials lists sample information. LC-grade methanol was purchased from Adamas-beta Co., Ltd. (Shanghai, China). LC–MS-grade formic acid was provided by Merck KGaA (Darmstadt, Germany). LC-grade acetonitrile was purchased from CNW Technologies GmbH (Duesseldorf, Germany). Water was purified using a Milli-Q 10 ultrapure water purification system (Merck KGaA).

HepG2 cells were purchased from the Cell Bank of the Chinese Academy of Science (Shanghai, China). Dulbecco’s modified eagle medium (DMEM), pancreatin, and penicillin–streptomycin solution were obtained from Thermo Fisher Scientific Co., Ltd. (Waltham, MA, USA). Fetal bovine serum (FBS) was purchased from Coriell Institute for Medical Research (Camden, NJ, USA). Dimethyl sulfoxide (DMSO) was obtained from MP Biomedicals Co., Ltd., (Shanghai, China). Phosphate-buffered saline (PBS) and 3-(4,5-dimethylthiazol-2-yl)-2,5-diphenyltetrazolium bromide (MTT) were obtained from Labgic Technology Co., Ltd. (Beijing, China). A 2-(3,6-diacetoxy-2,7-dichloro-9h-xanthen-9-yl) benzoic acid (DCFH-DA) probe was obtained from Abmole Bioscience Inc. (Houston, TX, USA), and 2,2′-azobis (2-amidinopropane) dihydrochloride (ABAP) was obtained from Rhawn Chemical Technology Co., Ltd. (Shanghai, China).

### 2.2. Sample Pretreatments

The coffee beans were milled with an IKA laboratory grinder (Staufen, Baden-Württemberg, Germany). A total of 500 mg of each sample was mixed to obtain a quality control (QC) sample. The extraction of coffee samples was performed according to a previous work by Asamenew et al. [9], with slight modifications. Specifically, each sample was weighted to 500 mg and dissolved using 5 mL of methanol:water:formic acid = 80:15:5 (*v*:*v*:*v*) in a 10 mL polypropylene centrifugation tube. The samples were then vortexed for 30 s and sonicated using an ultrasonic bath (Hechuang Ultrasonic, Shanghai, China) at 400 W and ambient temperature for 10 min. After centrifugation at 13,000 rpm and 4 °C for 10 min, the supernatants were filtered through a 0.22 µm syringe filter for further UPLC-MS analysis. All samples were prepared and tested in triplicate. 

A 0.5 mL aliquot of supernatant with an estimated concentration of 100 mg/mL was dried via constant nitrogen flow and re-dissolved in 3 mL of PBS for a further intracellular oxidative assay with HepG2 cells. The concentration of each sample used for the treatment of HepG2 cells was 16.7 mg/mL.

### 2.3. UPLC-MS Analysis

An ACQUITY ultra-performance liquid chromatograph hyphenated with a Xevo G2 quadrupole time-of-flight mass spectrometer (UPLC-QTOF-MS) (Waters Corporation, Milford, MA, USA) was used for sample analysis. The elution gradient was carried out with mobile phases A (0.1% formic acid in purified water, *v*/*v*) and B (0.1% formic acid in acetonitrile, *v*/*v*), and the flow rate was 0.4 mL/min. The linear gradient was performed as follows: 0–4 min, 5% B; 4–8 min, 5–25% B; 8–10 min, 25% B; 10–10.1 min, 25–100% B; 10.1–12 min, 100% B; 12–12.1 min, 100–5% B; and 12.1–14 min, 10% B. One 2 µL aliquot of the extract was injected into a Waters CORTECS C18 column (2.1 × 100 mm i.d.; 1.6 µm) with the column temperature at 40 °C. The electrospray ionization (ESI) source was set to the negative ionization mode, using source and desolvation temperatures of 120 and 500 °C, respectively. The capillary, sampling cone, and extraction cone voltages were 2.5 kV, 30 V, and 4.0 V, respectively. The flow rates of the cone and desolvation gases were 50 and 550 L/h, respectively. The data acquisition mode was MS^E^, which allowed for the simultaneous acquisition of both precursor and product ion information, enabling a more comprehensive structural elucidation of the analytes in complex samples. The mass acquisition range was 50–1500 Da. In the MS^1^ function, the collision energy was 6 eV, and fragment signals were produced under a collision energy ranging from 20 to 35 eV in MS^2^.

Compared to conventional data-dependent acquisition, where precursor ions are selectively fragmented based on their intensity and abundance, spurious peaks may have been generated in the MS^E^ mode because all fragment ions in both the low- and high-collision energy modes were collected. Consequently, to further prove the identities of the compounds using QTOF-MS, another independent verification was performed using a UPLC hyphenated with a Xevo TQS triple-quadrupole mass spectrometer (QqQ-MS) (Waters). The detailed parameters are given in Text S1 in the Supplementary Materials.

### 2.4. Data Processing

The acquisition of raw data was performed using MassLynx version 4.2 (Waters). Progenesis QI version 1.0 (Waters) was used for data pre-processing, including peak alignment, peak picking, and automatic compound identification using default settings. A quality control (QC) sample was automatically selected for peak alignment. All compounds were normalized according to a factor based on the reference run. The normalization factor was calculated by finding the mean of the log abundance ratios of the compounds that fall within the robust estimated limits [10]. Different adducts of the same compound were then grouped by deconvolution. A total of 1426 variables were detected. The phenolics database (PolyPhenols_PubChemID_v2013.24.11.01) from Pubchem [11], which contains 398 compounds, was applied for use in automatic compound identification in Progenesis QI. All normalized data were exported to MATLAB R2021b (The MathWorks, Natick, MA, USA) and MetaboAnalyst version 5.0 [12] for chemometrics modeling and statistical calculations. Significance between two data groups was tested using Student’s *t*-test. Autoscaling was used for data preprocessing. PCA, PLS-DA, and OPLS-DA were applied for modeling. The modeling performance was determined using the Q^2^ metric, an estimate of the predictive ability of the model. Q^2^ is calculated via cross-validation (CV) at different folds. The Q^2^ value is based on the evaluation of the prediction error between the predicted categorical variable ŷ and the actual output y. The prediction error is summed over all the samples (PRESS) (Equation (1)) and referred to the total sum of squares (TSS) (Equation (2)), and Q^2^ is then calculated using Equation (3). The closer Q^2^ is to 1, the better the prediction ability of the model is [13].

(1) $$PRESS=\sum_{i}\left(y_{i}-\hat{y_{i}}\right)$$

(2) $$TSS=\sum_{i}\left(y_{i}-\bar{y_{i}}\right)$$

(3) $$Q^{2}=1-\frac{PRESS}{TSS}$$

### 2.5. Cell Toxicity and Antioxidant Activity in HepG2 Cells

The HepG2 cells were cultured in a growth medium consisting of DMEM supplemented with 10% FBS and a 1% penicillin–streptomycin solution. The cells were maintained at 37 °C with 5% carbon dioxide, following the protocols described by Wolf et al. (Wolf & Liu, 2007). Cells within passages 10 to 20 were selected to ensure consistency and minimize any potential effects of long-term culturing.

#### 2.5.1. Cell Viability Assay

Cell viability was assessed using the MTT assay, following Liang et al.’s procedure [14] with slight modifications. Briefly, HepG2 cells in the logarithmic growth phase were seeded at a density of 1 × 10^3^ counts/well in a 96-well cell culture plate and pre-incubated at 37 °C in a 5% CO_2_ incubator for 24 h. Subsequently, 100 μL volumes of the 18 sample solutions, including a control group with PBS and a blank group with fresh medium simultaneously, were obtained, with each group prepared in triplicate. Except for the substitution of sample solutions with corresponding blank and control solutions, all other conditions remained identical to those used for the experimental group. After 24 h of treatment with two types of coffee products, 20 μL of the MTT solution (0.5 mg/mL) was added to each well and incubated for 5 h at 37 °C in a 5% CO_2_ incubator. The supernatant was then discarded, and 150 μL of DMSO was added to the solution, which was shaken for 10 min to dissolve the formazan using a shaker. The absorbance was determined at 490 nm and 570 nm using an Infinite M1000 PRO microplate reader (Tecan Group Ltd., Männedorf, Switzerland). The effects of the two different types of coffee products on the survival rate of the HepG2 cells were calculated according to Equation (4), where OD(E), OD(C), and OD(B) represent the absorbance values of the experimental group, control group, and blank group, respectively:

(4) $$Cell\;viability=\frac{OD\left(E\right)-OD(B)}{OD(C)-OD(B)}\times 100\%$$

#### 2.5.2. Cell Antioxidant Assay

The HepG2 cells were plated in sterile, all-black 96-well plates at a density of 6 × 10^3^ counts/well [15]. After the completion of cell culturing, the previous culture medium was removed from the wells. For the experimental group, 100 μL volumes of the sample solutions containing a DCFH-DA probe (25 μM) were added, while the control group received 100% DCFH-DA probe (25 μM in PBS) and the blank group received 100 μL of PBS. Except for the substitution of sample solutions with the corresponding blank and control solutions, all other conditions remained identical to those used for the experimental group. The cells were then placed in an incubator at 37 °C and 5% CO_2_ for 1 h. Subsequently, 100 μL of ABAP solution (600 μM) was added to both the experimental and control groups, while the blank group received 100 μL of PBS solution. The plates were immediately subjected to a real-time fluorescence enzyme-linked immunosorbent assay using a microplate reader (Tecan Group Ltd.). The measurement intervals were set at 5 min, and the fluorescence was measured continuously for 1 h. The excitation wavelength was 485 nm, and the emission wavelength was 538 nm. The method of calculating the cellular antioxidant activity (CAA) is presented in Equation (5), where ∫SA represents the area under the fluorescence–time curve formed by adding two different sources of astaxanthin, and ∫CA represents the area under the fluorescence–time curve formed by the blank group:

(5) $$CAA=\left(1-\frac{\int SA}{\int CA}\right)\times 100\%$$

## 3. Results and Discussion

### 3.1. Identification of Phenolic Acids

The chemical profiles of the QCs, including the BICs and the CBEs, were tentatively characterized. There were 1426 variables detected by Progenesis QI, with 81 compounds automatically identified (Table S2 in the Supplementary Materials). To obtain a more accurate identification, a second round of manual revision according to the MS^1^ and MS^2^ spectra extracted from the raw chromatogram of TOF-MS (Figure S1 in the Supplementary Materials), as well as the fragmentation patterns provided via QqQ-MS (Figure S2 and Table S3 in the Supplementary Materials) with confirmation from previous studies, was performed. A total of 25 compounds were further identified (Table 1) with relevant references (Table 2). 

CGAs were the most prevalent phenolics identified, consistent with a previous study [21]. CGAs are quinic acid esters with a trans-cinnamic acid moiety (such as caffeic, ferulic, and *p*-coumaric acid). Their content ranged from 3 to 12 g/100 g in green coffee (dry weight) [22], and they contribute to the bitter, sour, and astringent flavors of brewed coffee. The major subgroups of CGAs, including mono-caffeoylqiunic, di-caffeoylquinic, and feruloylquinc acids, account for approximately 98% of CGAs [23]. CGAs have been suggested to play a role in the defense mechanism against environmental aggressions and have been linked to anti-inflammatory, antiviral, and other health-promoting functionalities [24].

The identified compounds included four isomeric caffeoylquinic acids (CQAs, [M–H]^−^ = 353.0873), three feruloylquinic acids (FQAs, [M–H]^−^ = 367.1029), two CQA acid methyl esters (CQMs, [M–H]^−^ = 367.1029), six di-caffeoylquinic acids (di-CQAs, [M–H]^−^ = 515.1190), one feruloyl-caffeoylquinic acids (F-CQAs, [M–H]^−^ = 529.1346), four caffeoyl-quinolactones (CQLs, [M–H]^−^ = 335.0767), one feruloyl-quinolactone, (FQL, [M–H]^−^ = 529.1346), two di-caffeoyl-quinolactones (di-CQL, [M–H]^−^ = 497.1084), one caffeoyl-N-tryptophan ([M–H]^−^ = 365.1137), and one p-coumaroyl-N-tryptophan ([M–H]^−^ = 349.0923). The structures of the identified compounds were assigned based on complementary information obtained from the MS spectra, the relative hydrophobicity, the MS^1^ spectra, and fragmentation patterns in the MS^2^ spectra obtained in negative ion mode via QTOF-MS. The isomers were further confirmed based on previously published references, in which their health-promoting functions were discussed extensively as well (Text S2 in the Supplementary Materials and Section 3.2). It should be noted that all the identifications were tentative and require further investigation.

### 3.2. Difference in Phenolics Among Coffee Products

**Figure 1:**
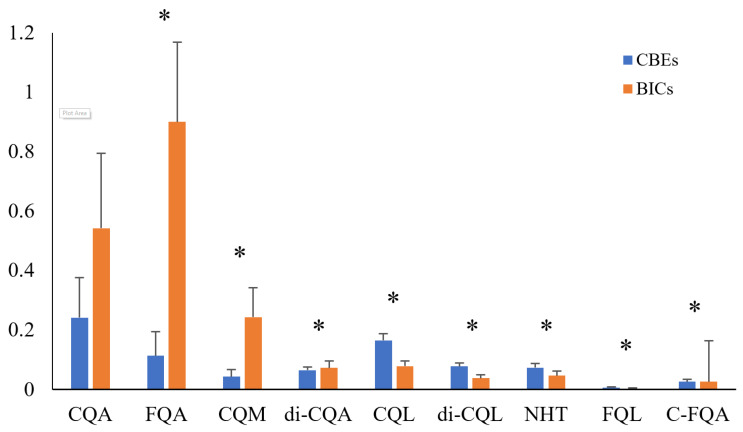
The contents of CGAs in BICs and CBEs. * indicates a significant difference in the compound groups (*p* < 0.01). CQAs and FQAs are the most abundant CGA subgroups in both BICs and CBEs. BICs generally show higher levels of CGAs compared to CBEs, indicating a potential concentration of these compounds during the instant coffee production process. The contents of CQLs, FQLs, and di-CQLs are slightly higher in coffee beans (CBEs) compared to BICs, which may contribute to the bitter taste in whole bean coffees.

The initial assessment of phenolics, as regards their overall abundance, may be helpful for extracting straightforward information for identifying coffee products. The substances in Table 1 were detected in all of the samples tested except for compound 12, 3,4-di-*O*-caffeoylquinic acid. Only a very low amount of compound 12 was detected in some of the black instant coffees (<0.15% of the total peak area for all samples). The normalized abundances of CGAs are shown in Figure 1. A heatmap of coffee chlorogenic acid profiles according to the compound group and individual samples is also given in Figure S3 in the Supplementary Materials. From these figures, we can see that the BICs had higher levels of CGAs, with CQAs and FQAs being the most abundant. This is potentially because the BIC products are extracted and concentrated to create a more potent coffee base during the production of modern instant coffee products. Brewing coffee may stimulate the release of phenols, leading to increased contents of bioactive substances, including various types of hydroxycinnamoyl derivatives. However, the contents of CQLs, FQLs, and di-CQLs in coffee beans were slightly higher than those in black instant coffees, which may contribute to the bitter taste of coffee beans. There were significant differences between all compound groups (*p* < 0.01), with the exception of CQAs, which returned a *p*-value of 0.54859. In summary, the overall abundance of CAG and its derivatives can offer a useful overview when identifying coffee products.

### 3.3. Classification of Black Instant Coffees and Coffee Bean Extracts Using Chemometrics

**Figure 2:**
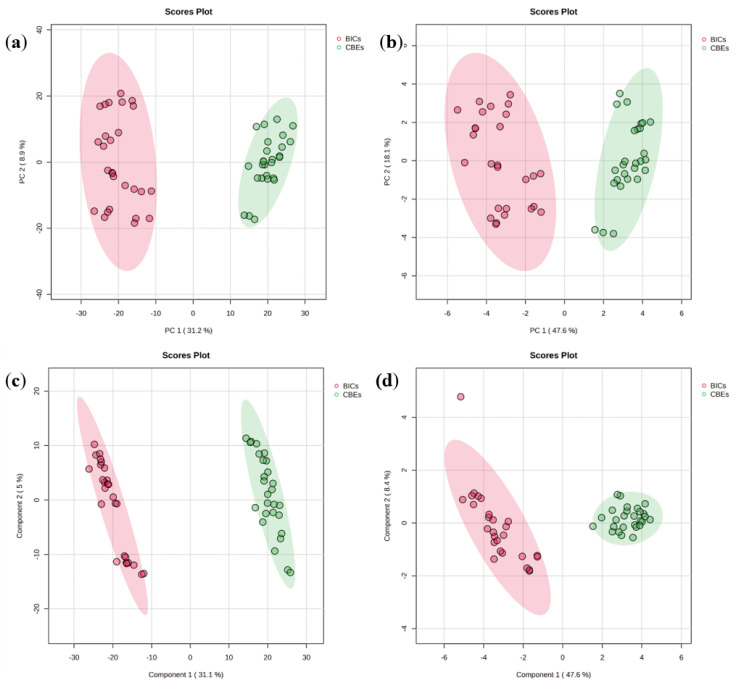
(**a**) PCA score plots with 1426 variables and (**b**) with 25 variables; (**c**) PLS-DA score plots with 1426 variables and (**d**) with 25 variables. The 95% confidence regions are displayed as color-shadowed ellipses. Both PCA and PLS-DA successfully classify BICs and CBEs, indicating that the phenolic profiles of these two coffee product types are significantly different. The identified CGAs contribute significantly to the classification models, highlighting their role as potential markers for distinguishing between BICs and CBEs. PLS-DA offers better separation compared to PCA, likely due to its supervised learning approach and focus on class separation.

While the abundances of different classes of compound profiles show some regularities among different coffee products, further multivariate modeling was necessary due to the complexity of the phenolic fingerprints. PCA and PLS-DA were first used to produce unbiased projections for all samples. Two datasets were used, i.e., the complete profile dataset of all 1426 variables identified and the simplified dataset using 25 manually reviewed compounds. The results show that CBEs and BICs were clearly distinguished along the PC1 orientation via PCA (Figure 2a,b). However, the first two principal components, PC1 and PC2, account for 40.1% of the total variance (Figure 2a), indicating the underlying complexity of the complete profile dataset. Therefore, a simplified dataset was applied to investigate the variable contribution of major components. The optimized PCA scores were plotted in Figure 2b, showing similar degrees of separation compared to the previous model using all available components. The PLS-DA score plots in Figure 2c,d also resulted in successful classification, with better degrees of separation than PCA, possibly because the PLS-DA is a mode of supervised modeling with advantages in achieving high predictive power on labeled training data. Since the complete profile dataset and the simplified dataset yielded similar results, it can be concluded that the 25 identified chlorogenic acids can effectively model the differences between coffee types.

Beyond the use of score plots to visually observe the classification of PLS-DA, more than two latent variables can be applied to yield an optimal identification. In addition, evaluation can be performed without human intervention in a fully automated, unbiased manner via chemometric validation techniques to determine modeling power. Using PLS-DA modeling to classify the BICs and CBEs resulted in 100% prediction accuracy, having employed the bootstrapped Latin-partition cross-validation approach [25] with 30 bootstraps and 10 partitions. The optimal number of latent variables was determined to be four, implying a relatively simple model. 

Two key metrics used in PLS-DA for model evaluation are the variable importance in projection (VIP) score and Q^2^. The VIP score is a measure used to assess the contribution of each variable in the model. It is calculated based on the weights of the PLS components and the variance of the predicted Y values. Variables with VIP scores greater than 1 (which is commonly used as a threshold) are considered to make a significant contribution to the model and are important in class separation. The VIP and loading plots of PLS-DA are consistent with the direct, univariate comparisons of phenolic profiles performed in Section 3.2. There were 14 substances identified as potentially important markers with VIPs > 1.0. The VIP scores of 4-C-muco-γ-Q indicated the highest importance at 1.4124, followed by 3-FQA at 1.3558, 5-FQA at 1.3153, pCoTry at 1.2967, 1,5-di-CQA at 1.2882, 3-C-*epi*-*γ*-Q at 1.2744, 3,4-di-C-*γ*-Q at 1.2433, 4-CQM at 1.2351, 4-FQA at 1.2140, 3-C-*γ*-Q at 1.1865, 3-CQM at 1.1757, 1,3-di-CQA at 1.1218, 3F-*epi*-*γ*-Q at 1.0452, and 4-CQA at 1.0097, indicating their substantial contributions to the model’s predictive ability. Specifically, in this model, 4-C-*muco*-*γ*-Q established the highest VIP value of 1.4124, suggesting it is a significant contributor to class separation. Other variables such as 3-FQA, 5-FQA, and pCoTry also showed VIP values above 1.3, highlighting their substantial influence on the model. Variables with VIP values above 1.2, such as 1,5-di-CQA and 3-C-*epi*-*γ*-Q, are also considered important. Those with VIP values just above 1, such as 3F-*epi*-*γ*-Q and 4-CQA, still contribute to the model, but to a lesser extent. These VIPs are consistent with the previous findings, shown in Figure 1, that some of the relative concentrations of the corresponding metabolites in BICs were higher than their counterparts in CBEs, including 3-FQA, 4-FQA, 5-FQA, 3-CQM, 4-CQM, 4-CQA, and 1,3-di-CQA. This result reveals that the CGAs in the BICs were higher than those in the CBEs. The variations in coffee processing approaches can result in the increased production of CGA and its derivatives within the BICs, which may induce notable alterations in both the nutritional composition and flavor profile of coffee. In summary, the profile of chlorogenic acid may differ between different types of coffee products and could be used as a precise quality metric for further evaluation.

Q^2^ is a measure of a model’s predictive ability. It is derived from cross-validation and indicates how well the model predicts new data. A Q^2^ value greater than 0.5 suggests that the model has good predictive performance. This model was evaluated using five-fold cross-validation, with a Q^2^ that reached 0.95361 using five components, indicating a satisfactory fit.

In summary, we applied PCA and PLS-DA to classify BICs and CBEs based on their phenolic profiles. The PCA score plots, utilizing both comprehensive and simplified datasets, can be used to clearly distinguish between CBEs and BICs, indicating distinct phenolic compositions. PLS-DA further enhanced classification accuracy, achieving 100% prediction accuracy and identifying 14 compounds with VIP scores > 1.0 as significant markers differentiating the two coffee types. This analysis confirms the unique phenolic signatures of BICs and CBEs, providing valuable insights for coffee product differentiation and quality assessment. This approach exemplifies the power of chemometrics in unraveling complex datasets, providing deeper insights into coffee chemistry and its functional properties, which is crucial for product development and quality control in the coffee industry.

### 3.4. Cell Toxicity and Antioxidant Activity

The phenolic profiles of different coffee products vary significantly, as evidenced by previous fingerprinting analyses. Consequently, it becomes essential to assess whether these differences impact the bioactivity of various coffee products. Two representative in vitro assays, the MTT assay and the CAA assay, were employed to determine the anticancer activities of phenolic compounds based on cell viability and to assess the ability of phenolic components to scavenge intracellular reactive oxygen species, respectively. The CAA comprehensively considers both the ability of antioxidant components to eliminate the reactive oxygen species within cells and their bioavailability. To assess the influence of BICs and CBEs on the survival rate of HepG2 cells, the samples were treated for a duration of 24 h, followed by the MTT assay.

**Figure 3:**
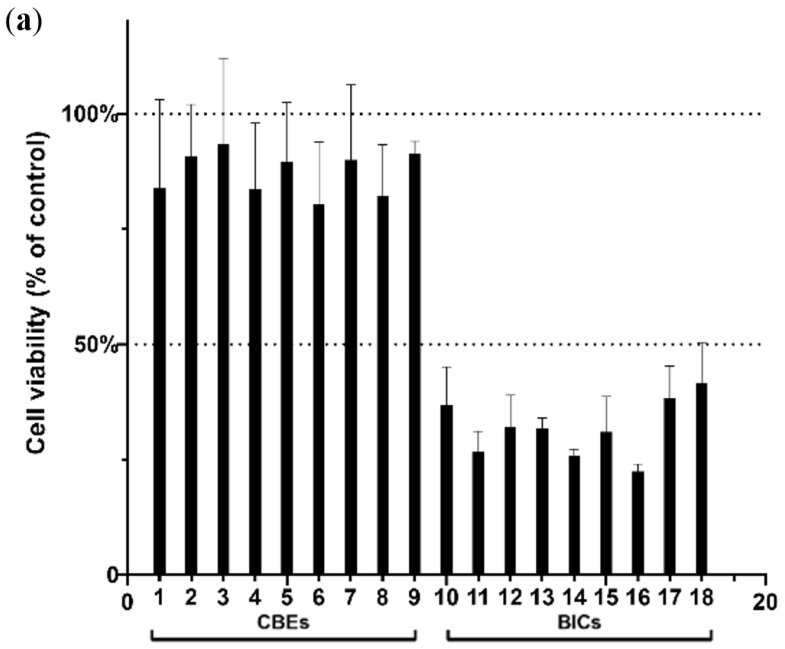

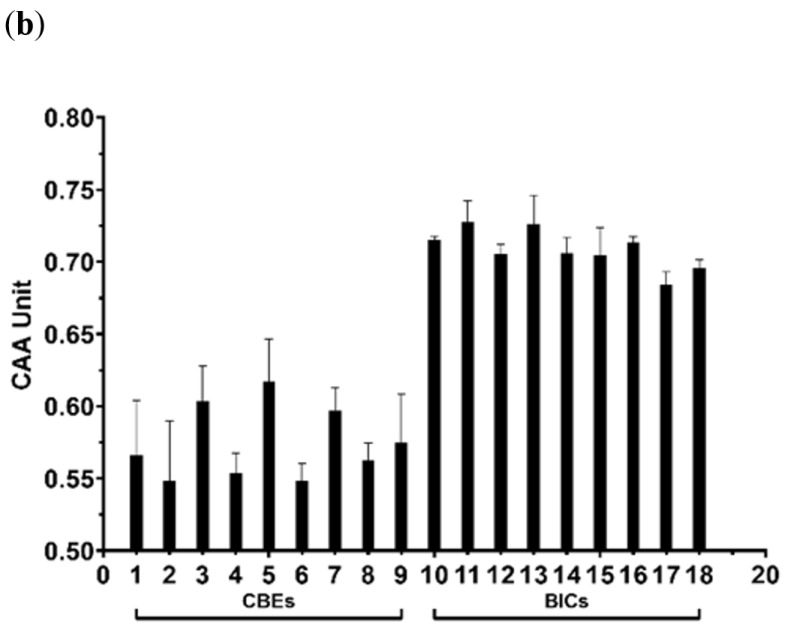
Effects of CBEs and BICs on (**a**) cell viability and (**b**) CAA of HepG2 cells. BICs exhibit significantly lower cell viability compared to CBEs, indicating potential cytotoxic effects; BICs exhibit significantly higher antioxidant activity compared to CBEs.

The results depicted in Figure 3a regarding the viability of the HepG2 cells between these two coffee product types are significantly different (*p* < 0.01). Specifically, the viability of the HepG2 cells treated with CBEs exhibited a higher rate of 80.45–93.45%, whereas the viability of those treated with the BICs showed a lower rate of 22.42–41.53%. These findings suggest that BICs might possess stronger cancer-preventive activity, whereas the CBEs are not significantly toxic to HepG2 cells. The lower viability rates observed in the BIC-treated cells suggest a potential cytotoxic effect, which may indicate a higher concentration of bioactive compounds in BICs that can impact cell survival. This result is particularly intriguing given the higher levels of CGAs and their derivatives found in BICs, as identified in our chemical profiling. These compounds are known for their antioxidant properties and have been implicated in the modulation of cellular responses, including those related to cancer cell lines. The differential cytotoxicity observed could be attributed to variations in phenolic composition between BICs and CBEs, which may be a result of the distinct processing methods to which each is subjected.

Figure 3b illustrates the dynamic changes in the ABAP-induced ROS oxidation of DCFH in HepG2 cells, revealing a significant difference between CBEs and BICs (*p* < 0.01). However, the results indicate that BICs exhibited stronger antioxidant activity compared to CBEs, with the CAA values for BICs approximately twice those of CBEs. The results of the in vitro assays align with the previous chemometrics findings that the concentrations of typical hydroxycinnamyl derivatives in the BICs were higher than those in the CBEs. In summary, these findings underscore the influence of the coffee brewing process on the antioxidant activities of these two coffee product types. This study suggests that BICs may yield better anti-cancer and antioxidant activities than CBEs. Compared with the results of cell viability, the antioxidant assay demonstrated that BICs exhibited stronger antioxidant activity compared to CBEs. This finding aligns with the higher CGA content in BICs, as these compounds are well-documented for their antioxidant capabilities. The ability of BICs to scavenge intracellular reactive oxygen species more effectively than CBEs suggests that the processing of instant coffee may concentrate these beneficial antioxidants. The increased antioxidant activity could have significant implications for the prevention of oxidative stress-related diseases, given the role of antioxidants in neutralizing harmful free radicals.

**Figure 4:**
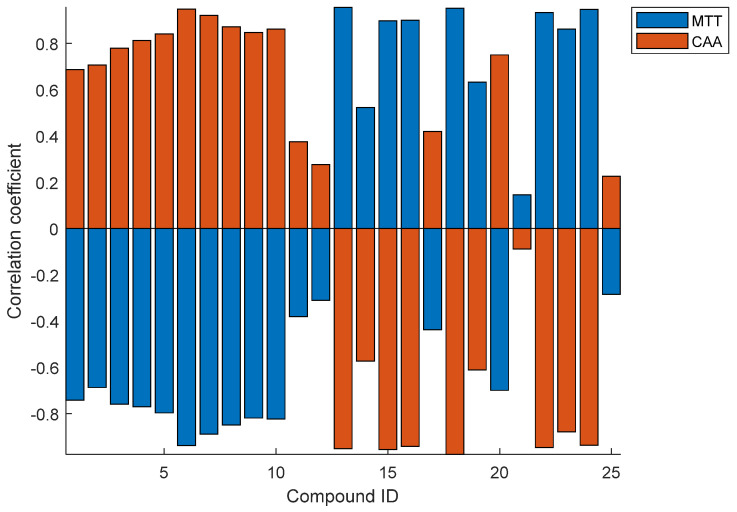
Correlation coefficients of MTT and CAA assays for each individual compound. Most compounds show strong correlations except for compounds 11, 12, 21, 25, etc. MTT and CAA are the complete opposite. For CAA, compounds 1–12, 17, 20, and 25 showed positive correlations.

Figure 4 presents the correlation coefficients of the MTT and CAA assays on each individual compound. Most compounds show strong correlations for both the MTT and CAA methods, indicating that the results of the two methods generally agree regarding bioactivities. There are specific compounds with weak correlation coefficients, such as 1,4-di-CQA and 3,4-di-CQA. On the other hand, the correlation coefficients for MTT and CAA are the complete opposite, which agrees with the results discussed previously. For CAA, compounds 1–12, 17, 20, and 25 showed positive correlations, with most of them being CQA, FQA, and CQM compounds. Overall, most of the components aligned with the results of bioactivity assays.

It is worth mentioning that the antioxidant effects, cell viability, and toxicity observed in our study are the cumulative results of the complex phenolic profiles present in BICs and CBEs. The synergistic effects of other phenolic compounds, such as ferulic acid and p-coumaric acid, may also contribute to the observed cell toxicity and antioxidant activity. The collective action of various polyphenolic compounds, rather than individual contributions, drives these biological outcomes. Additionally, the preparation method significantly influences the polyphenolic content, which in turn affects the overall bioactivity of coffee products. Our findings show that BICs contain higher levels of chlorogenic acids and their derivatives than CBEs, likely due to the concentration processes in instant coffee production. This underscores the impact of processing on the nutritional and functional attributes of coffee, suggesting that the choice of preparation method is crucial in determining the health benefits of coffee consumption.

## 4. Conclusions

In this study, comprehensive phenolic acidic profiles of BICs and CBEs were obtained and compared using UPLC combined with high-resolution QTOF-MS and QqQ-MS. A total of 25 compounds were identified. Afterward, chemometrics techniques including PCA, PLS-DA, and OPLS-DA were used to discriminate between different coffee products, differentiating BICs from CBEs.

Generally, more CGA and its derivatives were discovered in the BICs than in the CBEs. The results indicated that the phenolic compounds underwent complex and significant changes when raw coffee beans were processed into instant coffee, potentially impacting the nutritional value. Specifically, the BICs contained more CGAs, which impart a distinctive bitter and sour taste. The results also demonstrated the unique functional value of BICs, though the soluble coffees were generally considered less attractive than the whole bean coffees with respect to flavor. In a comparison of different chemometrics models, the PLS-DA and OPLS-DA models were more effective in classifying coffee bean types than PCA. The extracts of phenolic compounds from these two types of coffee products showed significant differences in the HepG2 cells. Since differences in the polyphenolic content depend on the type of sample and preparation method, processing techniques, such as roasting and instant coffee production, can significantly alter the polyphenolic composition, affecting the antioxidant properties and bioactivity of coffee products. In summary, this study reveals that phenols could act as possible markers for identifying different coffee products. On the other hand, various coffee products may exhibit unique phenolic profiles that impart distinct taste and flavor characteristics, suggesting an interesting relationship between the nutritional components and their functional properties. This study also suggests that comprehensive chemical and biochemical analyses, along with chemometrics techniques, provide valuable insights into the complex changes that occur in phenolic compounds during coffee processing. 

A key advantage of this work is the application of UPLC-MS to precisely profile phenolic compounds, which allows for a detailed characterization of coffee products. The integration of chemometric analysis enables the discrimination of complex datasets, providing a robust method for quality assessment and product differentiation. Furthermore, this study’s findings on the differential bioactivities of BICs and CBEs contribute to an understanding of coffee’s health effects. While our study offers a detailed comparison of BICs and CBEs, it is limited by its focus on a specific set of phenolic compounds and a single cell line for assessing bioactivity. This study does not account for the potential impacts of other coffee constituents, such as volatile compounds, which may also contribute to overall bioactivity. Additionally, the in vitro nature of the cell assays limits the direct extrapolation of these findings to in vivo conditions.

Future studies should focus on elucidating the synergistic or antagonistic interactions between various polyphenolic compounds in coffee, as these could significantly influence its overall bioactivity. Additionally, research should explore how different coffee preparation methods affect the polyphenolic content and profile and, consequently, the health benefits of the final product. This could involve comparing traditional brewing methods to modern techniques, such as cold brewing or nitrogen infusion, to determine their impacts on the polyphenolic composition and associated health effects. Future studies could also integrate variable selection to pinpoint phenolic markers linked to coffee’s health benefits and quality, enhancing our understanding and guiding coffee industry advancements.

## Figures and Tables

**Table 1:** Tentative identification of compounds from BICs and CBEs.

Peak ID.	RT (min)	Compound Abbreviation/Type	Formula	Exp. Mass [M–H]^−^	Accuracy (ppm)	Fragments Identified by UPLC-QTOF-MS
1	0.54	1-CQA	C_16_H_18_O_9_	353.0873	1.7	*135[caf-H-COO]^−^, 173[qui-H-H_2_O]^−^, *179[caf-H]^−^, *191[qui-H]^−^
2	0.72	3-CQA	C_16_H_18_O_9_	353.0873	5.9	*135[caf-H-COO]^−^, 179[caf-H]^−^, *191[qui-H]^−^
3	0.86	5-CQA	C_16_H_18_O_9_	353.0873	1.7	*135[caf-H-COO]^−^, *161[caf-H-H_2_O]^−^, *191[qui-H]^−^
4	1.22	4-CQA	C_16_H_18_O_9_	353.0873	0.3	*133[caf-H-H_2_O-CO]^−^, *135[caf-H-COO]^−^, 173[qui-H-H_2_O]^−^, *179[caf-H]^−^, *191[qui-H]^−^
5	1.35	3-CQM	C_17_H_20_O_9_	367.1029	3.5	134[caf-2H-COO]^−^, *135[caf-H-COO]^−^, *173[qui-H-H_2_O]^−^, 179[caf-H]^−^, *191[qui-H]^−^, 193[M+H_2_O-qui-H]^−^, *353[M-H-CH_3_]^−^,
6	1.59	3-FQA	C_17_H_20_O_9_	367.1029	4.9	*134[fer-H-CH_3_-COO]^−^, 173[qui-H-H_2_O]^−^, *175[fer-H-H_2_O]^−^, *191[qui-H]^−^, 193[fer-H]^−^
7	2.02	5-FQA	C_17_H_20_O_9_	367.1029	6	*134[fer-H-CH_3_-COO]^−^, *173[qui-H-H_2_O]^−^, *191[qui-H]^−^, *735[2M-H]^−^
8	2.21	4-CQM	C_17_H_20_O_9_	367.1029	4.6	*134[caf-2H-COO]^−^, 161[caf-H-H_2_O]^−^, *173[qui-H-H_2_O]^−^, 179[caf-H]^−^, 191[qui-H]^−^,
9	2.46	1,3-di-CQA	C_25_H_24_O_12_	515.1190	2.5	*161[caf-H-H_2_O]^−^, 173[qui-H-H2O]^−^, *191[qui-H]^−^
10	2.58	4-FQA	C_17_H_20_O_9_	367.1029	1.9	*134[fer-H-CH_3_-COO]^−^, 173[qui-H-H_2_O]^−^, *175[fer-H-H_2_O]^−^, 191[qui-H]^−^,
11	3.9	1,4-di-CQA	C_24_H_26_O_11_	515.1190	0.4	135[caf-H-COO]^−^, *161[caf-H-H_2_O]^−^, 173[qui-H-H_2_O]^−^, 179[caf-H]^−^, *191[qui-H]^−^
12	4.13	3,4-di-CQA	C_25_H_24_O_12_	515.1190	1.9	135[caf-H-COO]^−^, *173[qui-H-H_2_O]^−^, *191[qui-H]^−^
13	5.76	3-*O*-caffeoyl-*epi-γ*-quinide (3-C-*epi-γ*-Q)/CQL	C_16_H_16_O_8_	335.0767	5.7	*135[caf-H-COO]^−^, *161[caf-H-H_2_O]^−^, 173[qui-H-H_2_O]^−^, 179[caf-H]^−^, *191[qui-H]^−^
14	6.42	3,5-di-CQA	C_25_H_24_O_12_	515.1190	0.4	*161[caf-H-H_2_O]^−^, 173[qui-H-H_2_O]^−^, 179[caf-H]^−^, *191[qui-H]^−^, *353[M+H_2_O-caf-H]^−^
15	6.54	1,5-di-CQA	C_25_H_24_O_12_	515.1190	3.5	*161[caf-H-H_2_O]^−^, 173[qui-H-H_2_O]^−^, 179[caf-H]^−^, *191[qui-H]^−^, 353[M+H_2_O-caf-H]^−^
16	6.61	3-*O*-caffeoyl-*γ*-quinide (3-C-*γ*-Q)/CQL	C_16_H_16_O_8_	335.0767	0.6	*133[caf-H-H_2_O-CO]^−^, *135[caf-H-COO]^−^, *161[caf-H-H_2_O]^−^, *173[qui-H-H_2_O]^−^, 191[qui-H]^−^
17	6.70	4,5-di-CQA	C_25_H_24_O_12_	515.1190	0.6	135[caf-H-COO]^−^, 173[qui-H-H_2_O]^−^, 179[caf-H]^−^, *191[qui-H]^−^, *353[M+H_2_O-caf-H]^−^
18	6.81	4-*O*-caffeoyl-*muco-γ*-quinide (4-C-*muco-γ*-Q)/CQL	C_16_H_16_O_8_	335.0767	3.0	133[caf-H-H_2_O-CO]^−^, *135[caf-H-COO]^−^, *161[caf-H-H_2_O]^−^, 179[caf-H]^−^,
19	7.04	4-*O*-caffeoyl-*γ*-quinide (4-C-*γ*-Q)/CQL	C_16_H_16_O_8_	335.0767	4.0	133[caf-H-H_2_O-CO]^−^, 135[caf-H-COO]^−^, *161[caf-H-H_2_O]^−^, 173[qui-H-H_2_O]^−^, 179[caf-H]^−^, 191[qui-H]^−^
20	7.41	3C-5FQA/C-FQA	C_26_H_26_O_12_	529.1346	1.5	161[caf-H-H_2_O]^−^, *173[qui-H-H_2_O]^−^, *179[caf-H]^−^, *191[qui-H]^−^, *193[fer-H]^−^
21	7.56	caffeoyl-N-tryptophan (CTry)/HNT	C_20_H_18_N_2_O_5_	365.1137	3.6	135[caf-H-COO]^−^, 161[caf-H-H_2_O]^−^, 203[Try-H]^−^,
22	7.89	*p*-coumaroyl-*N*-tryptophan (pCoTry)	C_20_H_18_N_2_O_4_	349.1188	18.0	173[qui-H-H_2_O]^−^, *191[qui-H]^−^, *203[Try-H]^−^
23	7.97	Isomers of 3-*O*-feruloyl-*γ*-quinide (3F*-epi-γ-*Q)/FQL	C_17_H_18_O_8_	349.0923	4.9	173[qui-H-H_2_O]^−^, 175[fer-H-H_2_O]^−^, 191[qui-H]^−^
24	9.73	3,4-*O*-dicaffeoyl-*γ*-quinide (3,4-di-C-*γ*-Q)/di-CQL	C_25_H_22_O_11_	497.1084	0.4	161[caf-H-H_2_O]^−^, 173[qui-H-H_2_O]^−^, 179[caf-H]^−^, 191[qui-H]^−^, *335
25	10.45	4,5-*O*-dicaffeoyl-*muco-γ*-quinide (4,5-di-C*-muco-γ-*Q)/di-CQL	C_25_H_22_O_11_	497.1084	3.0	161[caf-H-H_2_O]^−^, 173[qui-H-H_2_O]^−^, 179[caf-H]^−^, 191[qui-H]^−^, *335

RT, retention time; CQA, caffeoylquinic acid; CQM, caffeoylquinic acid methyl ester; FQA, feruloylquinic acid; di-CQA, di-caffeoylquinic acid; C-FQA, caffeoyl-feruloylquinic acid; CQL, chlorogenic acid lactone; FQL, feruloyl-quinolactone; di-CQL, di-caffeoylquinic acid lactone; HNT, hydroxycinnamoyl-N-tryptophan; Qui, quinic acid; Caf, caffeic acid; Fer, ferulic acid. * fragments consistently detected in UPLC-QqQ-MS.

**Table 2:** Structural information of identified compounds.

Class	Name and Abbreviation	References
CGAs	1/3/5/4-*O*-caffeoylquinic acid (1-CQA, 3-CQA, 5-CQA, 4-CQA)	[9,16,17]
CGAs	3/5/4-*O*-feruloylquinic acid (3-FQA, 5-FQA, 4-FQA)	[9,16,17,18]
CGAs	1,3/1,4/3,4/3,5/1,5/4,5-di-*O*-caffeoylquinic acid (1,3-di-CQA, 1,4-di-CQA, 3,4-di-CQA, 3,5-di-CQA, 1,5-di-CQA, 4,5-di-CQA)	[9,17,18,19]
CGAs	3-*O*-feruloyl, 4-*O*-caffeoylquinic acid (3F,4CQA)	[9,16,17,18]
CGA derivatives	4/5-*O*-caffeoylquinic acid methyl (4-CQM, 5-CQM)	[9,17]
Cinnamoyl quinides	Isomers of 3-*O*-caffeoyl-*γ*-quinide (3-C-*γ*-Q, 3C*-epi-γ-*Q)	[9,20]
Cinnamoyl quinides	Isomers of 3-*O*-feruloyl-*γ*-quinide (3F*-epi-γ-*Q)	[9,20]
Cinnamoyl quinides	3,4/4,5-di-*O*-caffeoylquinide (di-CQL)	[9]
Cinnamoyl-amino acid conjugates	Caffeoyl-*N*-tryptophan (CTry)	[9,17]
Cinnamoyl-amino acid conjugates	*p*-coumaroyl-*N*-tryptophan (pCoTry)	[9,17]

## Data Availability

The original contributions presented in the study are included in the article/Supplementary Materials, further inquiries can be directed to the corresponding author.

## Supplementary Materials

The following supporting information can be downloaded at: https://www.mdpi.com/article/10.3390/foods13244016/s1, Text S1. Instrument settings for UPLC-QqQ-MS analysis; Text S2. Additional compound identification information of phenolic acids. Table S1. Sample information list. Table S2. Identified compounds through database searching by Progenesis QI. Table S3. Structural representation of phenolic derivatives in coffee. Figure S1. BPI chromatogram of UPLC/Q-TOF-MS. Figure S2. Schema of the general fragmentation pattern Figure S3. Heatmap of coffee chlorogenic acid profiles. Figure S4. MS1 and MS2 spectra of CQAs (1) 1-CQA, (2) 3-CQA, (3) 5-CQA, and (4) 4-CQA. Figure S5. MS1 and MS2 spectra of FQAs. (1) 3-FQA, (2) 5-FQA, and (3) 4-FQA. Figure S6. MS1 and MS2 spectra of CQMs. (1) 5-CQM (2) 4-CQM. Figure S7. MS1 and MS2 spectra of diCQAs: (1) 1,3-diCQA, (2) 1,4-diCQA, (3) 3,4-diCQA, (4) 3,5-diCQA, (5) 1,5-diCQA, and (6) 4,5-diCQA. Figure S8. MS1 and MS2 spectra of 3C-5FQA. Figure S9. MS1 and MS2 spectra of cinnamyl quinolacton (1) 3-C-epi-γ-Q, (2) 3-C-γ-Q, (3) 4-C-muco-γ-Q, (4) 4-C-γ-Q. Figure S10. MS1 and MS2 spectra of cinnamyl quinolacton: 3F-epi-γ-Q. Figure S11. MS1 and MS2 spectra of diCQLs (1) 3,4-diCQL (2) 4,5-diCQL. Figure S12. MS1 and MS2 spectra of HNTs (1) caffeoyl-N-tryptophan (2) p-coumaroyl-N-tryptophan.
