# Supplementary material for: Chemometric Classification and Bioactivity Correlation of Black Instant Coffee and Coffee Bean Extract by Chlorogenic Acid Profiling
Source: Foods. 2024 Dec 12;13(24):4016. doi: 10.3390/foods13244016 (PMC11726881; doi:10.3390/foods13244016)
Supplement: Supplementary file 1 [file foods-13-04016-s001.zip › Supplementary Material L20.pdf]

Supplementary Material

Chemometric Classification and Bioactivity Correlation of Black Instant Coffee and Coffee Bean Extract by Chlorogenic Acid Profiling

Yumei Chen [1], Wei Yu [1], Yuge Niu [1], Wenchen Li [1], Weiying Lu [1,]* and Liangli (Lucy) Yu [2]

[1] Institute of Food and Nutraceutical Science, Department of Food Science and Technology, School of Agriculture and Biology, Shanghai Jiao Tong University, Agriculture and Biology Building, 800 DongChuan Road, Shanghai 200240, China; c18324939875@163.com (Y.C.); yu-wei@sjtu.edu.cn (W.Y.);
yugeniu@sjtu.edu.cn (Y.N.); wensjtu@sjtu.edu.cn (W.L.)

[2] Department of Nutrition and Food Science, University of Maryland, College Park, MD 20742, USA; lyu5@umd.edu

* Correspondence: weiying.lu@sjtu.edu.cn; Tel.: +(86)-21-3420-4041

Table of Contents

Text S1. Instrument settings for UPLC-QqQ-MS analysis

Text S2. Additional compound identification information of phenolic acids

Table S1. Sample information list

Table S2. Identified compounds through database searching by Progenesis QI. (Given in a separate spreadsheet due to its size.)

Table S3. Structural representation of phenolic derivatives in coffee

Figure. S1. BPI chromatogram of UPLC/Q-TOF-MS

Figure. S2. Schema of the general fragmentation pattern

Figure. S3. Heatmap of coffee chlorogenic acid profiles

Figure. S4. MS^1 and MS^2 spectra of CQAs (1) 1-CQA, (2) 3-CQA, (3) 5-CQA, and (4) 4-CQA.

Figure. S5. MS^1 and MS^2 spectra of FQAs. (1) 3-FQA, (2) 5-FQA, and (3) 4-FQA.

Figure. S6. MS^1 and MS^2 spectra of CQMs. (1) 5-CQM (2) 4-CQM.

Figure. S7. MS^1 and MS^2 spectra of diCQAs: (1) 1,3-diCQA, (2) 1,4-diCQA, (3) 3,4-diCQA, (4) 3,5-diCQA, (5) 1,5-diCQA, and (6) 4,5-diCQA.

Figure. S8. MS^1 and MS^2 spectra of 3C-5FQA.

Figure. S9. MS^1 and MS^2 spectra of cinnamyl quinolacton (1) 3-C-*epi*-*γ*-Q, (2) 3-C-*γ*-Q, (3) 4-C-*muco*-*γ*-Q, (4) 4-C-*γ*-Q.

Figure. S10. MS^1 and MS^2 spectra of cinnamyl quinolacton: 3F-*epi*-*γ*-Q.

Figure. S11. MS^1 and MS^2 spectra of diCQLs (1) 3,4-diCQL (2) 4,5-diCQL.

Figure. S12. MS^1 and MS^2 spectra of HNTs (1) caffeoyl-N-tryptophan (2) *p*-

coumaroyl-N-tryptophan.

Text S1. Instrument settings for UPLC-QqQ-MS analysis

A Waters ACQUITY ultraperformance liquid chromatography combined with a Xevo TQS triple quadrupole mass spectrometry (UPLC-QqQ-MS) (Waters, Milford, Massachusetts, USA) was used for sample analyses. The optimized 14-min gradient was carried out with mobile phase A (0.1% formic acid in purified water, v/v) and B (0.1% formic acid in acetonitrile, v/v), and the flow rate was 0.4 mL/ min. An aliquot of 2 μL extracts were injected into a Waters Acquity UPLC Cortex C18 column (2.1 × 100 mm i.d.; 1.6 μm) with the column temperature at 40 °C. The linear gradient was performed as follows: 0–4 min, 5% B; 4–8 min, 5–25% B; 8–10 min, 25% B; 10–10.1 min, 25-100% B; 10.1–12 min, 100% B; 12–12.1 min, 100–5% B; 12.1–14 min, 10% B. The ESI source parameters were: capillary voltage, 2.5 kV (negative mode); extractor voltage, 4.0 V; sampling cone voltage, 30 V; temperature and flow rate of the desolvatation gas (Nitrogen), 500°C and 550 L/h^{-1}; cone and nebuliser gas (N2) flow rate, 150 L/h^{-1} and 7.0 bar respectively; and source block temperature, 120°C. The collision energy in MS mode was 4 eV, in MS/MS mode was 30 eV.

Acquisition in both MS scan and product ion scan modes was performed in centroid mode monitoring from m/z 50 to 1000 or to a m/z value slightly higher than the precursor ion respectively; 1 scan/s; and interscan delay of 0.05 s.

Text S2. Additional compound identification information of phenolic acids

1. Caffeoylquinic acid (CQA)

CQAs are one important group with various biological activities of natural products in herbal medicines and food plants. Various biological activities have been reported for CQAs such as anti-human immunodeficiency virus (HIV), anti- respiratory syncytial virus (RSV) antimutagenicity, antioxidant activities for CQAs combined with relatively low toxicity and side effects (Li, Y. L., But, P. P. H., & Ooi, V. E. S. (2012). Antiviral activity and mode of action of caffeoylquinic acids from *Schefflera heptaphylla* (L.) Frodin. *Antiviral Research, 68*(1), 1-9. https://doi.org/10.1002/bmc.1685). Farah et al. (Farah, A., Monteiro, M. C., Calado, V., Franca, A. S., & Trugo, L. C. (2006). Correlation between cup quality and chemical attributes of Brazilian coffee. *Food Chemistry, 98*(2), 373-380. https://doi.org/10.1016/j.foodchem.2005.07.032) observed that higher levels of caffeoylquinic acids (predominantly 5-CQA) and their oxidation products are associated with poor cup quality and with the Rio off-flavor. CQA has diverse isomers such as 1-, 3-, 4-, and 5-CQAs, with different retention and MS fragmentation. They could yield characteristic fragments at m/z = 353, 191, 179, 173, 161, and 135 by loss of H, caffeic residue, quinic residue, caffeic residue and H_2O, quinic residue, and H_2O, respectively. 1-CQA was the first one to elution referred to the previous study (Asamenew, G., Kim, H. W., Lee, M. K., Lee, S. H., Lee, S., Cha, Y. S., Lee, S. H., Yoo, S. M., & Kim, J. B. (2019). Comprehensive characterization of hydroxycinnamoyl derivatives in green and roasted coffee beans: A new group of methyl

hydroxycinnamoyl quinate. *Food Chemistry-X, 2*, Article 100033. https://doi.org/10.1016/j.fochx.2019.100033). Besides, dehydrated ion of quinic acid at m/z = 173 was only formed by CGA with a cinnamoyl group bonded to the quinic moiety at position 4, as described by other studies (Clifford, M. N., Johnston, K. L., Knight, S., & Kuhnert, N. (2003). Hierarchical scheme for LC-MS^n identification of chlorogenic acids. *Journal of Agricultural and Food Chemistry, 51*(10), 2900-2911. https://doi.org/10.1021/jf026187q). The fragment of m/z = 173 was detected in **peak 4** by both UPLC-QTOF-MS and QqQ-MS, but not detected simultaneously in other peaks. Peak 4 was identified as 4-CQA. Besides, their elution order of the isomers allowed an unambiguous identification of the four CQA according to previous studies using QqQ MS/MS (Alonso-Salces, R. M., Guillou, C., & Berrueta, L. A. (2009). Liquid chromatography coupled with ultraviolet absorbance detection, electrospray ionization, collision-induced dissociation and tandem mass spectrometry on a triple quadrupole for the on-line characterization of polyphenols and methylxanthines in green coffee beans. *Rapid Communications in Mass Spectrometry, 23*(3), 363-383. https://doi.org/10.1002/rcm.3884). Therefore, **peaks 1**, **2**, **3**, **4** were identified as 1-, 3-, 5-, and 4-CQAs, respectively, as was shown in **Figure. S4**.

2. Feruloylquinic acids (FQA)

FQAs are quinic acid esters with ferulic acids moieties. The CQA isomers were always higher than FQA isomers in commercial brewed coffees (Gao, C., Tello, E., & Peterson, D. G. (2023). Identification of compounds that enhance bitterness of coffee

brew. *Food Chemistry, 415*, 135674. https://doi.org/10.1016/j.foodchem.2023.135674). Extracted MS chromatograms at m/z = 367.1029 revealed 5 peaks. Three FQAs were assigned by the presence or absence of caffeic acid residue at m/z = 179. The fragmentation pattern of FQA was similar to CQA. There were characteristic base peaks ion in these three isomers: m/z = 134 [ferulic acid-H-CH_3-COO] $^-$, 173 [quinic acid-H_2O-H]$^-$, 175 [ferulic acid-H_2O-H]$^-$, m/z = 191 [quinic acid-H] $^-$, 193 [ferulic acid-H]$^-$, 175 [ferulic acid-H_2O-H]$^-$.The three isomers yielded different base peaks in the MS^2 channel and elution order, which allowed the unambiguous identification of the three FQA. **Peak 10** showed a low intensity compared with **peaks 6** and **7**. **Peak 6** was identified as 3-FQA, **peak 7** as 5-FQA, and **peak 10** as 4-FQA as was shown in **Figure. S5**.

3. Caffeoylquinic acids methyl ester (CQM)

CQMs are relatively uncommon coffee esters. They show biological activities like antioxidant, anti-stress, anti-aging and inhibit collagenase, reverse transcriptase and DNA polymerase (Teramachi, F., Koyano, T., Kowithayakorn, T., Hayashi, M., Komiyama, K., & Ishibashi, M. (2005). Collagenase inhibitory quinic acid esters from *Ipomoea pes-caprae*. *Journal of Natural Products, 68*(5), 794-796. https://doi.org/10.1021/np0500631). Two CQM isomers at m/z = 367.1029 were detected in the extracted MS chromatograms. The same fragment at m/z = 179 [Caffeic acid-H]$^-$ at different isomers confirmed they were CQMs rather than FQAs. The MS^2 fragments of QqQ-MS showed the similar pattern. Moreover, the dehydrated

ion of caffeic acid at m/z = 161 provided further evidence for the existence of CQM. Considering that the chemical bond between the cinnamoyl moiety and quinic acid at position 4 is the strongest, and at position 3 the weakest, **peaks 5** and **8** were equivocally assigned 3- and 4-CQM, respectively (**Figure. S6**).

4. Di-caffeoylquinic acids (di-CQA)

Di-CQAs were common CGAs coupled with CQA, different vegetables were reported abundant in caffeoylquinic acids and di-caffeoylquinic acids, such as bay leaf, mustard, celery, rosemary, collard greens and chicory with different di-CQA concentrations (Meinhart, A. D., Damin, F. M., Caldeirao, L., de Jesus Filho, M., da Silva, L. C., Constant, L. d. S., Filho, J. T., Wagner, R., & Godoy, H. T. (2019). Study of new sources of six chlorogenic acids and caffeic acid. *Journal of Food Composition and Analysis, 82*, 103244. https://doi.org/10.1016/j.jfca.2019.103244). Six di-CQAs were detected in extracted MS chromatograms at m/z = 515.1190, two caffeic acids were connected to quinic acid, which offered the higher intensity at m/z = 179 [caffeic acid-H]$^-$. The quinic moiety [quinic-H]$^-$ at m/z = 191 were detected with high relative abundance (RA) in **peak 9** (100%), **13** (100%), **14** (100%), **18** (100%), **19** (84%) and **21** (53%). Different isomers were estimated depending on the strength of the chemical bond. The order of chemical bond between the cinnamoyl moiety and quinic acid were position 4 stronger than 5 higher than 3 higher than 1. Therefore, **peaks 9** and **21** could be assigned as 1,3- and 4,5-CQA. The fragments of m/z = 161 and 191 were detected in these six isomers by QqQ-MS. The results accorded with Clifford et al. (Clifford, M.

N., Knight, S., Surucu, B., & Kuhnert, N. (2006). Characterization by LC-MS^n of four new classes of chlorogenic acids in green coffee beans: Dimethoxycinnamoylquinic acids, diferuloylquinic acids, caffeoyl-dimethoxycinnamoylquinic acids, and feruloyl-dimethoxycinnamoylquinic acids. *Journal of Agricultural and Food Chemistry, 54*(6), 1957-1969. https://doi.org/10.1021/jf0601665), the sequence of elution for six di-CQA isomers were determined as 1,3- <<< 1,4- < 3,4- < 3,5- = 1,5- (co-eluted) << 4,5- CQAs. **Peaks 9**, **11, 12, 14, 15** and **17** were identified according to this order. The MS^1 and MS^2 spectra of diCQA were shown in **Figure. S7**.

5. Caffeoyl-feruloylquinic acid (C-FQA)

The extracted MS chromatograms at m/z = 529.1346 have been identified as caffeoyl-feruloylquinic acid. Of the six C-FQAs previously identified in green coffee beans (Clifford, M. N., Johnston, K. L., Knight, S., & Kuhnert, N. (2003). Hierarchical scheme for LC-MS^n identification of chlorogenic acids. *Journal of Agricultural and Food Chemistry, 51*(10), 2900-2911. https://doi.org/10.1021/jf026187q), only one was detected in this study due to the possible limited sensitivity. The higher relative abundance of fragments at m/z = 179 [caffeic acid-H]$^-$ and 161 [caffeic acid-H-H_2O]$^-$ suggests that caffeic acid was bonded to position 1. The highest relative abundance was at m/z = 173 due that the cinnamoyl moiety being bonded to position 4 of quinic acid. **Peak 20** has been identified as 3-C, 5-FQA, as shown in **Figure. S8**.

6. Chlorogenic acid lactone (CQL)

CQLs were bitter-tasting lactones that can be generated in the high-temperature coffee roasted process through transesterification, epimerization, and lactonization reactions involving CQA and FQA (Asamenew, G., Kim, H. W., Lee, M. K., Lee, S. H., Lee, S., Cha, Y. S., Lee, S. H., Yoo, S. M., & Kim, J. B. (2019). Comprehensive characterization of hydroxycinnamoyl derivatives in green and roasted coffee beans: A new group of methyl hydroxycinnamoyl quinate. *Food Chemistry-X, 2*, Article 100033. https://doi.org/10.1016/j.fochx.2019.100033). These lactones exhibit hypoglycemic effects in rats, and their potential biological activities have been linked to their effects on brain functioning, specifically the antagonism of the *μ* opioid receptor and adenosine transporter (de Paulis, T., Commers, P., Farah, A., Zhao, J., McDonald, M. P., Galici, R., & Martin, P. R. (2004). 4-Caffeoyl-1,5-quinide in roasted coffee inhibits [^{3}H]naloxone binding and reverses anti-nociceptive effects of morphine in mice. *Psychopharmacology, 176*, 146-153. https://doi.org/10.1007/s00213-004-1876-9.). Four CQLs were identified through extracted MS chromatograms (**Figure. S9**). Quinolactone has a similar structure to quinic acid, with a lactone structure between the carboxyl and hydroxyl groups at positions 3, 4, and 5 in quinic acid. In fact, six lactones, namely 3-*O*-caffeoyl-*epi*-*γ*-quinide (3-C-*epi*-*γ*-Q), 5-*O*-caffeoyl-*muco*-*γ*-quinide, 3-*O*-caffeoyl-*γ*-quinide (3-C-*γ*-Q), 4-*O*-caffeoyl-*muco*-*γ*-quinide (4-C-*muco*-*γ*-Q), 5-*O*-caffeoyl-*epi*-*δ*-quinide, and 4-*O*-caffeoyl-*γ*-quinide (4-C-*γ*-Q), have been confirmed by Frank et al. (Frank, O., Blumberg, S., Krümpel, G., & Hofmann, T. (2008). Structure determination of 3-O-caffeoyl-epi-γ-quinide, an orphan bitter lactone in roasted coffee.

Journal of Agricultural and Food Chemistry, 56(20), 9581-9585. https://doi.org/10.1021/jf802210a). This study identified four isomers of 3C-*epi*-*γ*-Q at **peaks 13**, **16**, **18**, and **19**, following their elution order. Other isomers were not detected in this study, possibly due to their low concentration. The corresponding compositions of these isomers remained unclear, due to the limited data. They were ambiguously identified as 3-C-*epi*-*γ*-Q, 3-C-*γ*-Q, 4-C-*muco*-*γ*-Q and 4-C-*γ*-Q.

7. Feruloyl-quinolactone (FQL)

Peak 23 was identified as an isomer of 3-*O*-feruloyl-*γ*-quinide. The $[M-H]^-$ peak at m/z = 349.0923 and the ferulic residue peak at m/z = 193 provide evidence for the existence of FQL, which has been previously confirmed in another study (Asamenew, G., Kim, H. W., Lee, M. K., Lee, S. H., Lee, S., Cha, Y. S., Lee, S. H., Yoo, S. M., & Kim, J. B. (2019). Comprehensive characterization of hydroxycinnamoyl derivatives in green and roasted coffee beans: A new group of methyl hydroxycinnamoyl quinate. Food Chemistry-X, 2, Article 100033. https://doi.org/10.1016/j.fochx.2019.100033). The accurate spectra corresponding to **peak 23** can be found in **Figure. S10**.

8. Di-caffeoylquinic acid lactone (di-CQL)

The di-CQLs contribute mainly to the bitter-tasting of coffee. The 3,4-*O*-dicaffeoyl-*γ*-quinide, 3,5-*O*-dicaffeoyl-*epi*-*δ*-quinide, and 4,5-*O*-dicaffeoyl-*muco*-*γ*-quinide were found to be generated from the corresponding *O*-dicaffeoylquinic acids. Sensory studies have shown that the bitterness threshold of these lactones vary

between 9.8 and 180 μmol/L (Frank, O., Blumberg, S., Krümpel, G., & Hofmann, T. (2008). Structure determination of 3-O-caffeoyl-epi-γ-quinide, an orphan bitter lactone in roasted coffee. *Journal of Agricultural and Food Chemistry, 56*(20), 9581-9585. https://doi.org/10.1021/jf802210a). Peak 30 and 31 in extracted MS chromatograms at m/z = 497.1084 had been tentatively identified as 3,4-*O*-dicaffeoyl-*γ*-quinide (3,4-di-C-*γ*-Q) and 4,5-*O*-dicaffeoyl-*muco*-*γ*-quinide (4,5-di-C-*muco*-*γ*-Q). The MS^2 spectrum revealed the detection of CQL at m/z = 335, caffeic residue at m/z = 179, and hydrated caffeic residue at m/z = 161. The same fragments at m/z = 173 indicated that one of the CQL molecules was connected at position 4. The elution time difference among the isomers and the peak intensity analysis by Frank et al. (Frank, O., Blumberg, S., Krümpel, G., & Hofmann, T. (2008). Structure determination of 3-O-caffeoyl-epi-γ-quinide, an orphan bitter lactone in roasted coffee. *Journal of Agricultural and Food Chemistry, 56*(20), 9581-9585. https://doi.org/10.1021/jf802210a) allowed for the tentative identification of **peaks 24** and **25** as 3,4-di-C-*γ*-Q and 4,5-di-C-*muco*-*γ*-Q as shown in **Figure. S11**, respectively.

9. Hydroxycinnamoyl-N-tryptophan (HNT)

Cinnamoyl-amino acid conjugates may affect the aroma and flavor of the beverage (Rodrigues, N. P., & Bragagnolo, N. (2013). Identification and quantification of bioactive compounds in coffee brews by HPLC–DAD–MS^n. *Journal of Food Composition and Analysis, 32*(2), 105-115. https://doi.org/https://doi.org/10.1016/j.jfca.2013.09.002.). **Peaks 21** and **22**

correspond to caffeoyl-*N*-tryptophan and *p*-coumaroyl-*N*-tryptophan, respectively, which are formed by the attachment of a cinnamoyl moiety to tryptophan. The fragmentation pattern of these compounds, showing fragment at m/z = 203 $[\text{tryptophan-H}]^-$ both in QTOF-MS and QqQ-MS. The spectra of UPLC-QTOF-MS was shown in **Figure. S12**.

Table S1. Sample information list

No.	Type	Brands	Origin	Degree of roasting
1	Coffee beans	A	Guatemala	Medium
2	Coffee beans	A	Brazil	Medium
3	Coffee beans	B	Portugal	Dark
4	Coffee beans	B	Portugal	Medium
5	Coffee beans	C	China	Dark
6	Coffee beans	C	China	Dark
7	Coffee beans	C	China	Medium
8	Coffee beans	C	China	Dark
9	Coffee beans	D	China	Dark
10	Black instant coffee	E	Netherlands	N. A.
11	Black instant coffee	F	Japan	N. A.
12	Black instant coffee	F	Japan	N. A.
13	Black instant coffee	G	England	N. A.
14	Black instant coffee	D	Columbia	N. A.
15	Black instant coffee	D	Columbia	N. A.
16	Black instant coffee	B	N. A.	N. A.
17	Black instant coffee	B	N. A.	N. A.
18	Black instant coffee	H	China	N. A.

N. A., not available. Brands were shown anonymously in codenames.

Table S2. Identified compounds through database searching by Progenesis QI. (Given in a separate spreadsheet due to its size.)

Table S3. Structural representation of phenolic derivatives in coffee

Class	Name and abbreviation	Structure representation	Substitution group 1	Substitution group 2
CGAs	1/3/5/4-*O*-caffeoylquinic acid (1-CQA, 3-CQA, 5-CQA, 4-CQA) (example as 1-CQA)	R_A-R_1	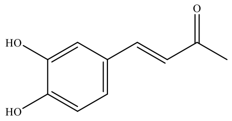	-H
CGAs	3/5/4-*O*-feruloylquinic acid (3-FQA, 5-FQA, 4-FQA) (example as 1-FQA)	R_A-R_1	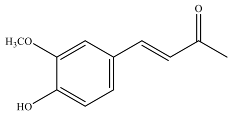	-H
CGAs	1,3/1,4/3,4/3,5/1,5/4,5-di-*O*-caffeoylquinic acid (1,3-di-CQA, 1,4-di-CQA, 3,4-di-CQA, 3,5-di-CQA, 1,5-di-CQA, 4,5-di-CQA) (example as 1,3-di-CQA)	R_B-R_1-R_1	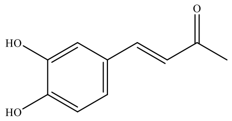	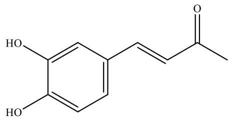
CGAs	3-*O*-feruloyl, 4-*O*-caffeoylquinic acid (3F,4CQA) (example as 1C,3FQA)	R_B-R_1-R_1	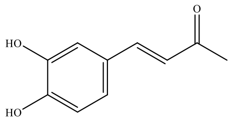	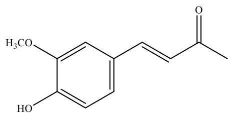
CGA derivatives	4/5-*O*-caffeoylquinic acid methyl (4-CQM, 5-CQM) (example as 1-CQM)	R_A-R_1-R_1	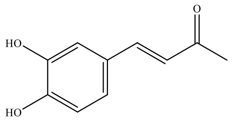	$-CH_3$
Cinnamoyl quinides	Isomers of 3-*O*-caffeoyl-*γ*-quinide (3C-*epi*-*γ*-Q)	R_C-R_1	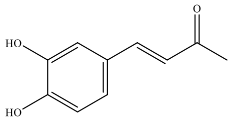	N.A.
Cinnamoyl quinides	Isomers of 3-*O*-feruloyl-*γ*-quinide (3F-*epi*-*γ*-Q)	R_C-R_1	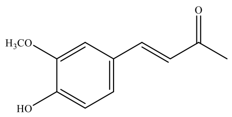	N.A.
Cinnamoyl quinides	3,4/4,5-di-*O*-caffeoylquinide (di-CQL)	R_C-R_1-R_2	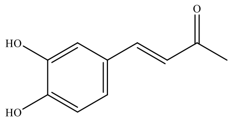	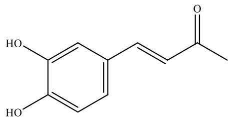
Cinnamoyl-amino acid conjugates	Caffeoyl-*N*-tryptophan (CTry)	R_E-R_1	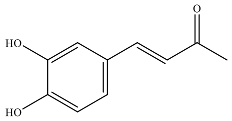	N.A.
Cinnamoyl-amino acid conjugates	*p*-coumaroyl-*N*-tryptophan (pCoTry)	R_E-R_1	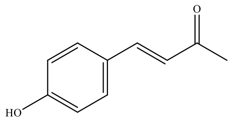	N.A.

The general fragmentation patterns of phenolic compounds are presented in **Fig. S2**.

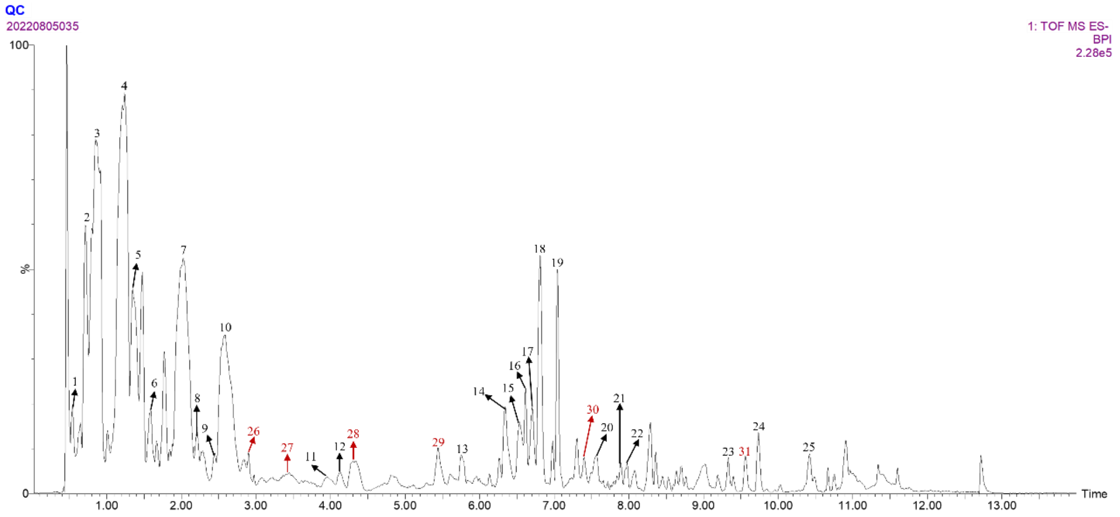

Figure. S1. BPI chromatogram of UPLC/Q-TOF-MS. Red peaks indicates new discovered compounds.

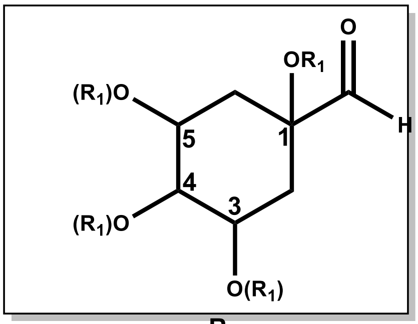

R_A

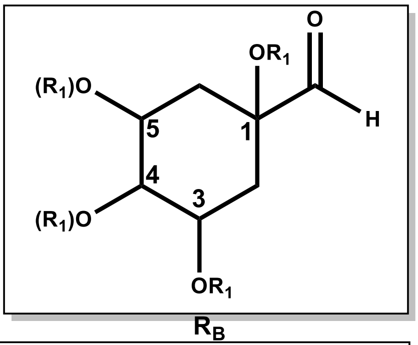

R_B

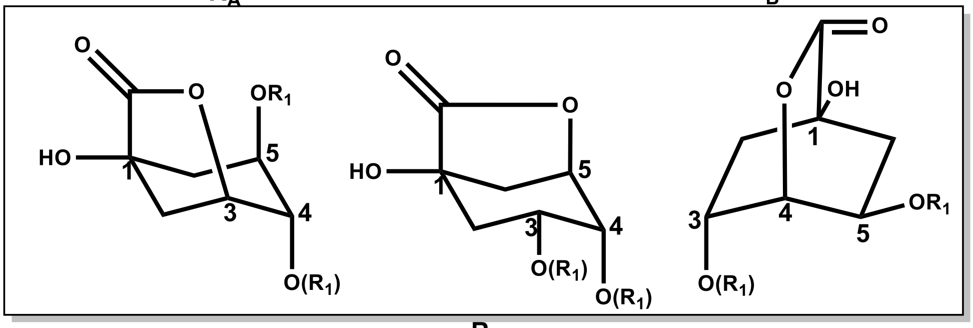

R_C

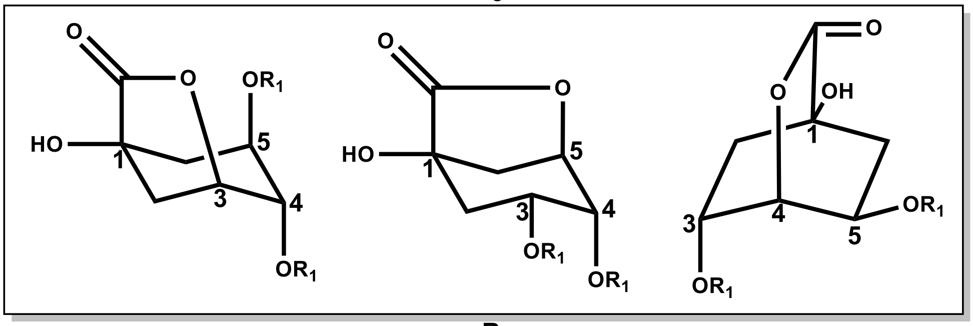

R_D

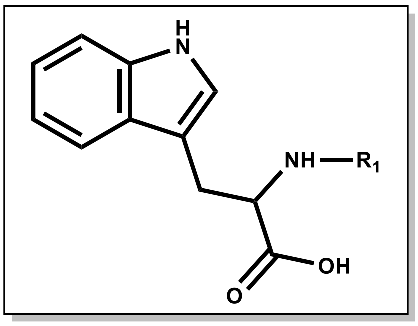

R_E

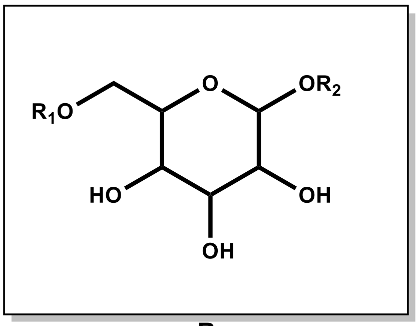

R_F

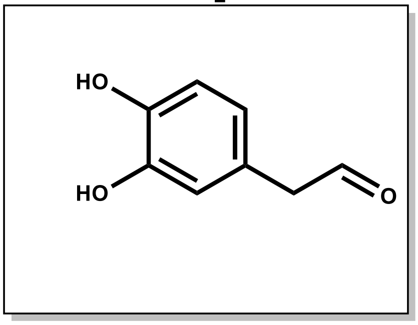

R_G

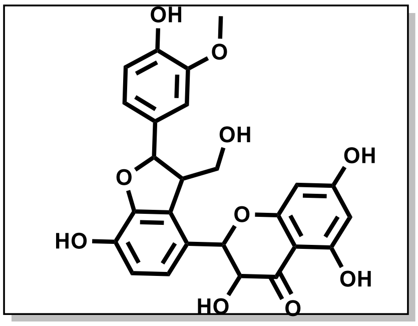

R_H

Figure. S2. Schema of the general fragmentation pattern. The structure of R_C and R_D were obtained from Frank et al., 2008.

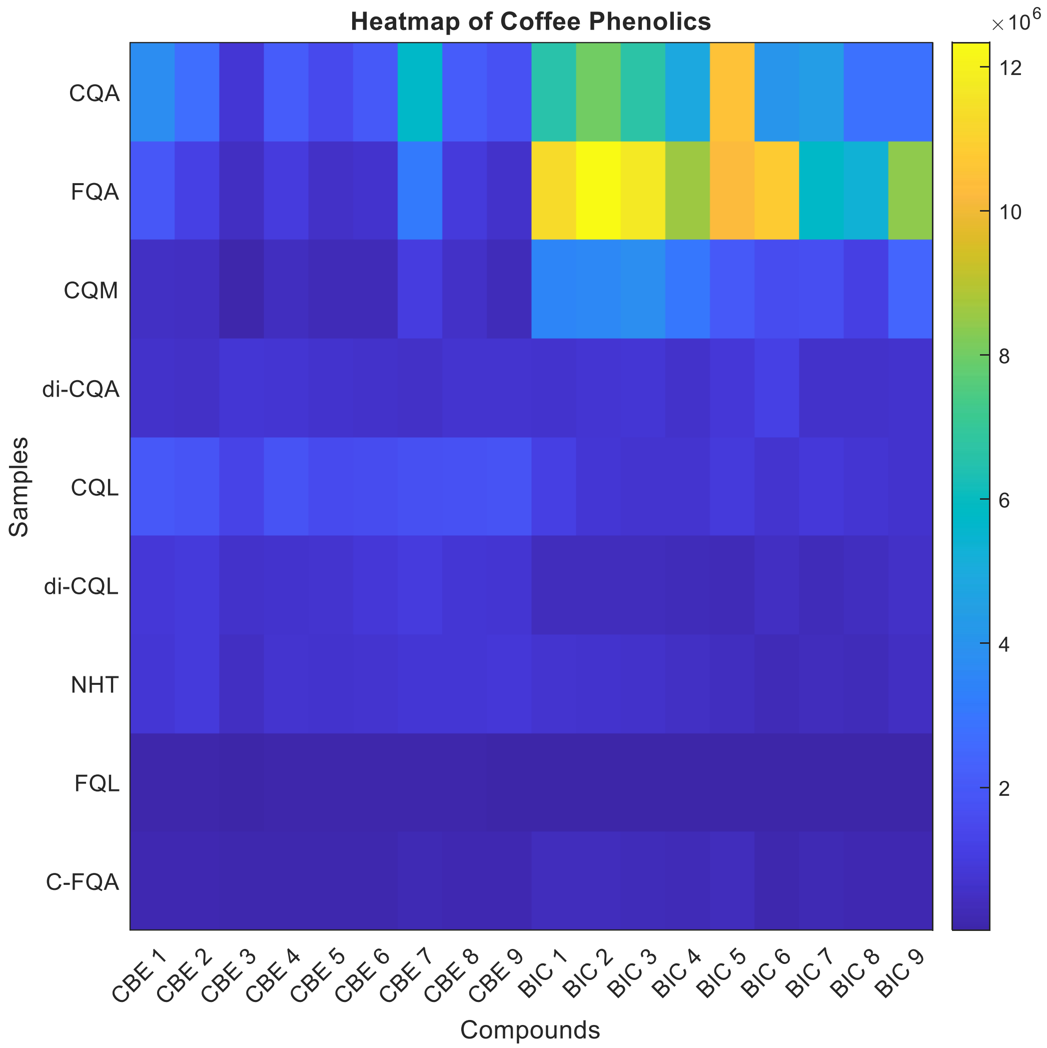

Figure. S3. Heatmap of coffee chlorogenic acid profiles.

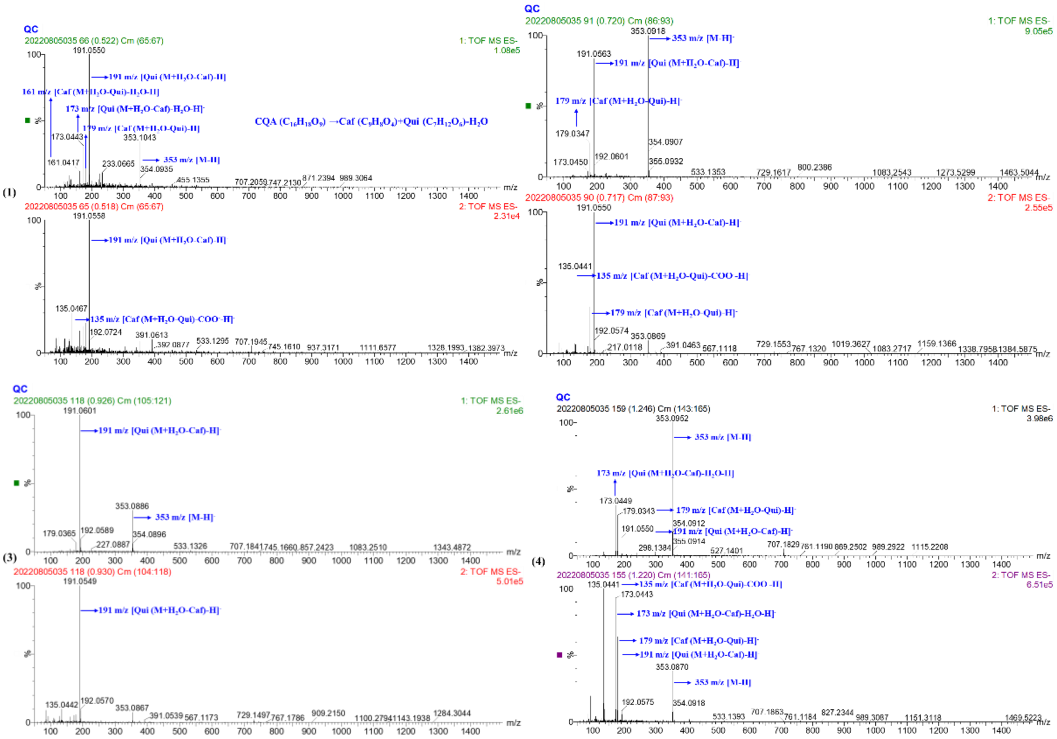

Figure. S4. MS^1 and MS^2 spectra of CQAs (1) 1-CQA, (2) 3-CQA, (3) 5-CQA, and (4) 4-CQA. See abbreviations in **Table 1**.

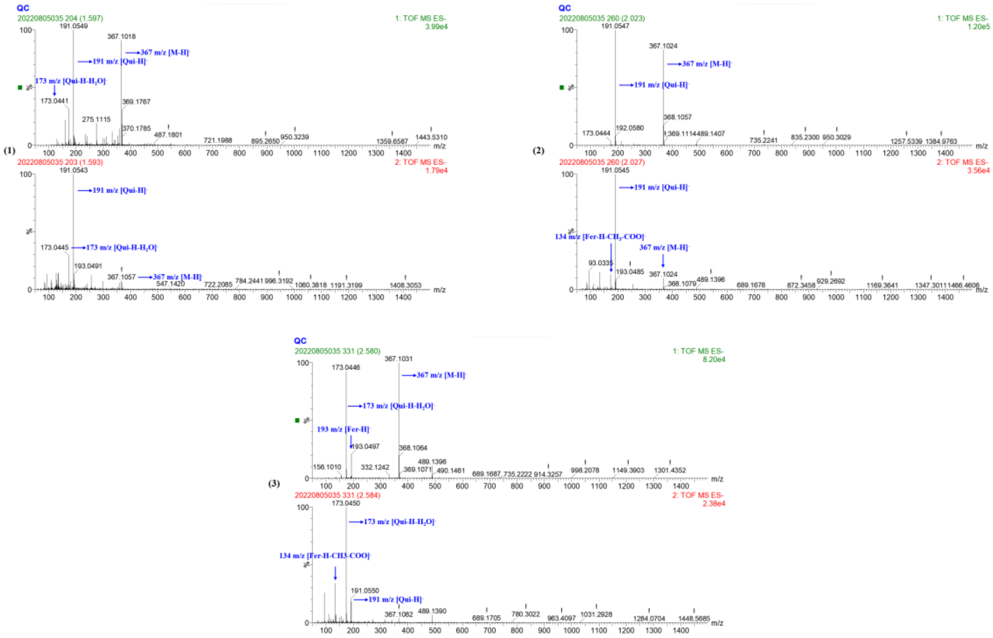

Figure. S5. MS^1 and MS^2 spectra of FQAs. (1) 3-FQA, (2) 5-FQA, and (3) 4-FQA. See abbreviations in **Table 1**.

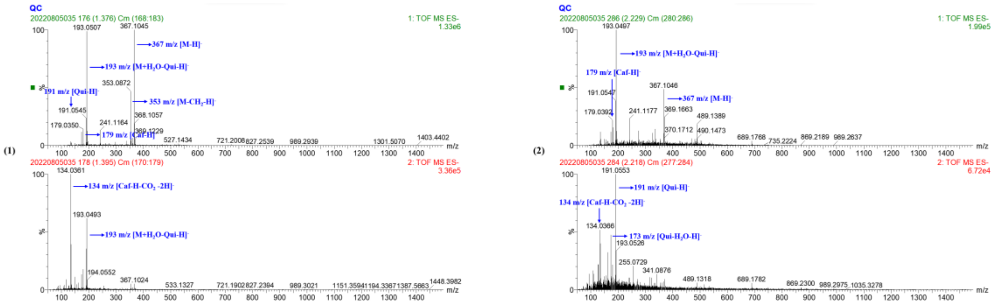

Figure. S6. MS^1 and MS^2 spectra of CQMs. (1) 5-CQM (2) 4-CQM. See abbreviations in **Table 1**.

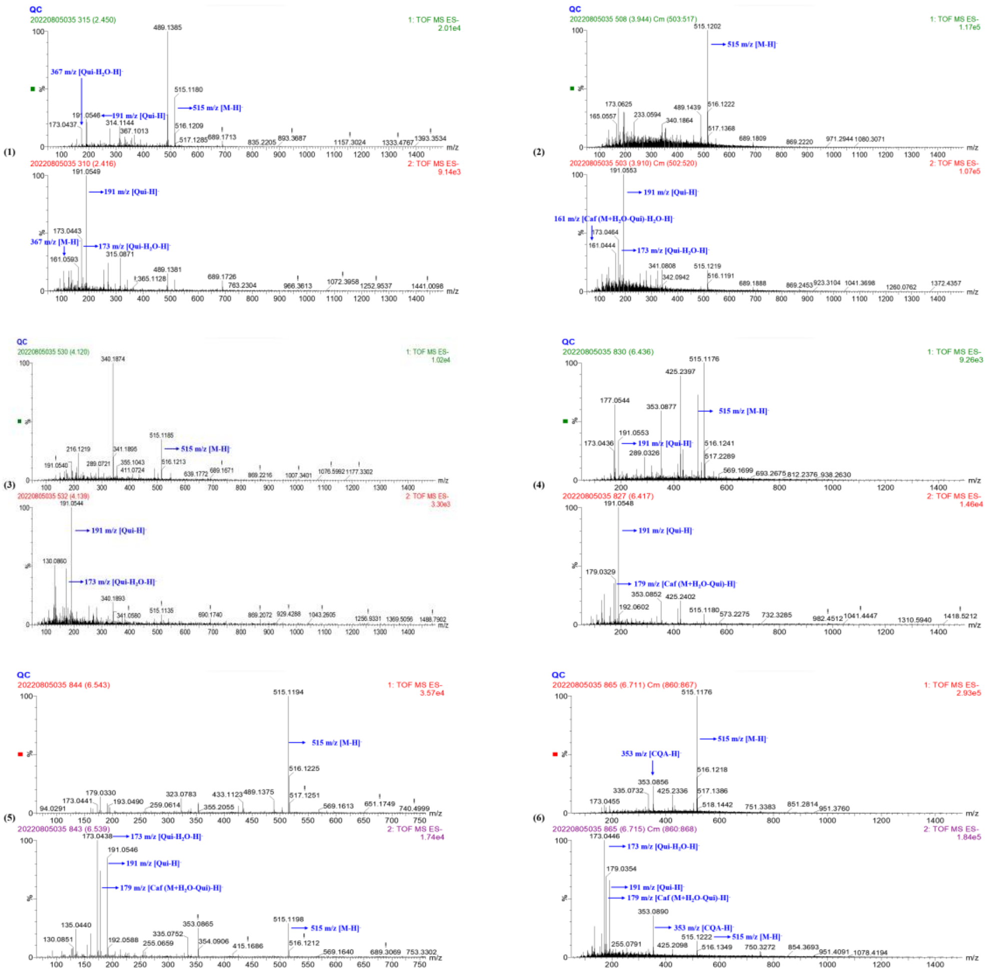

Figure. S7. MS[1] and MS[2] spectra of diCQAs: (1) 1,3-diCQA, (2) 1,4-diCQA, (3) 3,4-diCQA, (4) 3,5-diCQA, (5) 1,5-diCQA, and (6) 4,5-diCQA. See abbreviations in **Table 1**.

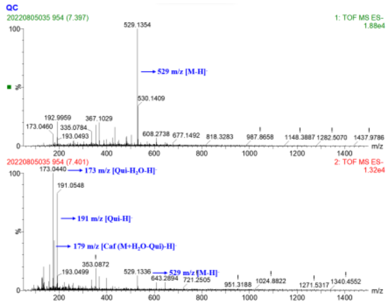

Figure. S8. MS^1 and MS^2 spectra of 3C-5FQA. See abbreviations in **Table 1**.

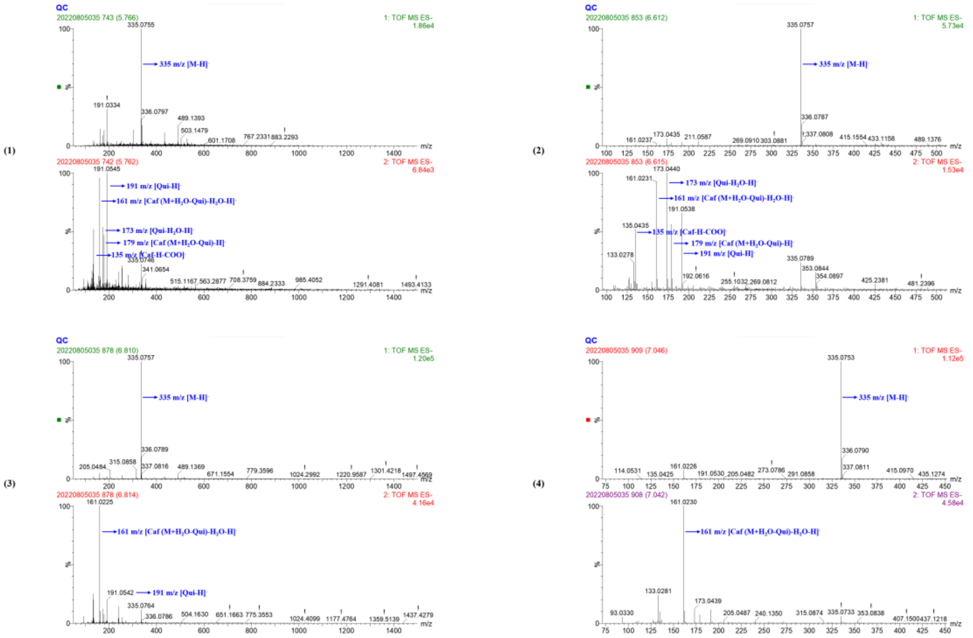

Figure. S9. MS^1 and MS^2 spectra of cinnamyl quinolacton (1) 3-C-*epi*-*γ*-Q, (2) 3-C-*γ*-Q, (3) 4-C-*muco*-*γ*-Q, (4) 4-C-*γ*-Q. See abbreviations in **Table 1**.

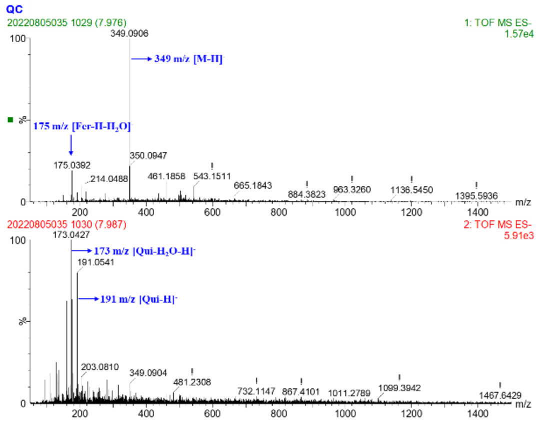

Figure. S10. MS[1] and MS[2] spectra of cinnamyl quinolacton: 3F-*epi*-γ-Q. See abbreviations in **Table 1**.

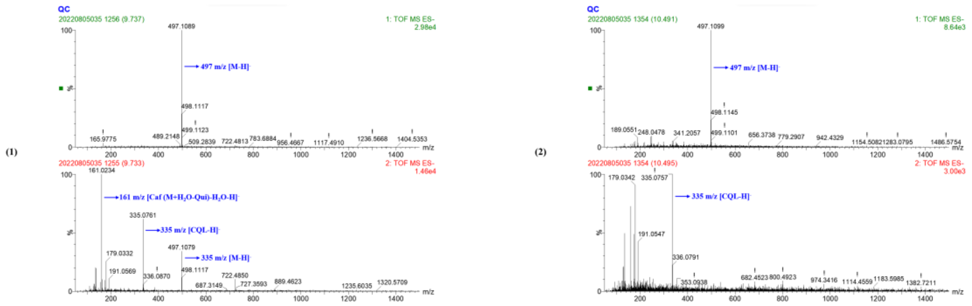

Figure. S11. MS^1 and MS^2 spectra of diCQLs (1) 3,4-diCQL (2) 4,5-diCQL. See abbreviations in **Table 1**.

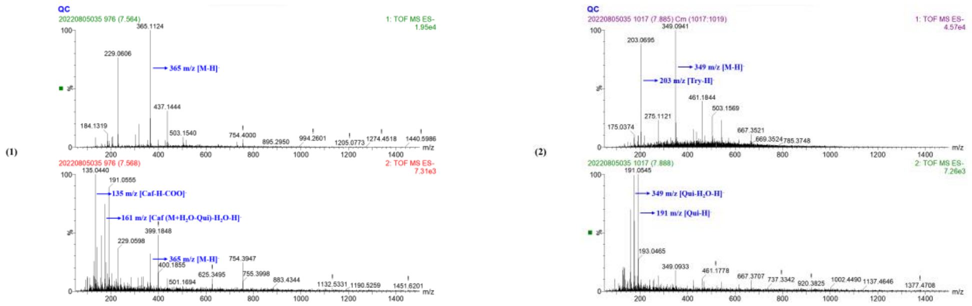

Figure. S12. MS[1] and MS[2] spectra of HNTs (1) caffeoyl-N-tryptophan (2) *p*-coumaroyl-N-tryptophan. See abbreviations in **Table 1.**
